# Phylogenetic Inference Using Cytochrome C Oxidase Subunit I (COI) in the Poultry Red Mite, *Dermanyssus gallinae* in the United Kingdom Relative to a European Framework

**DOI:** 10.3389/fvets.2020.00553

**Published:** 2020-08-21

**Authors:** Eleanor Karp-Tatham, Tatiana Küster, Athanasios Angelou, Elias Papadopoulos, Alasdair J. Nisbet, Dong Xia, Fiona M. Tomley, Damer P. Blake

**Affiliations:** ^1^Pathobiology and Population Sciences, The Royal Veterinary College, Brookmans Park, United Kingdom; ^2^Laboratory of Parasitology and Parasitic Diseases, School of Veterinary Medicine, Aristotle University of Thessaloniki, Thessaloniki, Greece; ^3^Vaccines, Pentlands Science Park, Moredun Research Institute, Penicuik, United Kingdom

**Keywords:** *Dermanyssus gallinae*, poultry red mite, cytochrome C oxidase subunit 1, diversity, chickens

## Abstract

The poultry red mite *(Dermanyssus gallinae*), an obligatory blood feeding ectoparasite, is primarily associated with laying hens where it is estimated to cause losses of ~€231 million per annum to European farmers. Moderate to high infestation levels result in negative impacts on hen welfare, including increased cannibalism, irritation, feather pecking, restlessness, anemia, and mortality. Acaricides are currently the prevailing method of population control for *D. gallinae*, although resistance against some classes of acaricide has been widely reported. The development of resistance highlights a growing need for research into alternative control methods, including the development of a suitable and effective vaccine. Understanding the genetic structure of *D. gallinae* populations can support improved management of acaricide resistance and sustainability of future vaccines, but limited data are currently available. The aim of this study was to characterize *D. gallinae* isolates from Europe, targeting the cytochrome c oxidase subunit 1 (COI) gene to gain an insight into population structure and genetic diversity of currently circulating mites. *Dermanyssus gallinae* isolates were collected from Albania, Belgium, Croatia, Czech Republic, Denmark, France, Greece, Italy, the Netherlands, Portugal, Romania, Slovenia, Turkey and the United Kingdom. Genomic DNA was extracted from individual adult *D. gallinae* mites and a 681bp fragment of the COI gene was amplified and sequenced. Phylogenetic analyses of 195 COI sequences confirmed the presence of multiple lineages across Europe with 76 distinct haplotypes split across three main haplogroups and six sub-haplogroups. Importantly there is considerable inter- and intra-country variation across Europe, which could result from the movement of poultry or transfer of contaminated equipment and/or materials and husbandry practices.

## Introduction

*Dermanyssus gallinae* (de Geer) is an obligatory blood feeding ectoparasite (1). A worldwide distribution has been reported for *D. gallinae* with a high percentage of affected premises in European countries including Serbia, the Netherlands, Denmark, Romania, France, Poland, Italy and the United Kingdom (UK) (2–7). In the UK, for example, between 60 and 85% of commercial egg laying systems are reported to be infested (4, 5). *Dermanyssus gallinae* causes significant economic loss to the European poultry industry, with a cost estimate of ~€231 million per annum (8) that is attributed to higher feed conversion ratios, production losses and the cost of control (9). Annual costs for the UK alone are estimated at €3 million (9). Affected birds have decreased egg production, irritation and, in severe infections, anemia leading to death (10). Research by Kilpinen et al. (11) on the influence of *D. gallinae* infections on laying hen health showed a reduction in weight gain in young birds when comparing mite-infested hens to hens without *D. gallinae* infestation. After 100 days infected birds still had a significantly lower weight (11). *D. gallinae* may also play a role in the transmission of other pathogenic agents and may act as a reservoir for some pathogens for example *Salmonella enterica* var Enteritidis (12–14). *Dermanyssus gallinae* infestation in Europe is becoming an increasing problem due in part to the banning of some chemical treatments and to changes in husbandry practices such as the use of enriched cages that help to facilitate the survival and spread of the parasite (9).

*Dermanyssus gallinae* displays some plasticity in terms of host specificity and in addition to avians some isolates have been shown to be capable of feeding to some extent on mammals, including horses, rodents, and humans (12). Studies on the genetic diversity of *D. gallinae* have focused on several targets including cytochrome c oxidase subunit 1 (COI) (10, 15, 16), 16S rDNA (17, 18), and the rDNA internal transcribed spacer (ITS) regions (15–17, 19, 20). Overall, results have indicated that populations of mites show patterns of genetic diversity both within and between international borders. This is exemplified in a study by Chu et al. (19), who studied the genetic diversity of COI amongst *D. gallinae* found within Japan and discovered that some populations mites from Japan were genetically related to those from Europe (19). This is supported by similar evidence from Korea (21). Roy et al. (17, 18) investigated species limits of several isolates of *D. gallinae* from various regions of Europe. They demonstrated species variation of <9% for COI and, based on further analysis, concluded that *D. gallinae* represents a complex of hybridized lineages, possibly species, from a total of 35 haplotypes (17). Studies with ITS sequences have been less informative, revealing limited or no variation, although differences have been observed between mite groups collected from domestic chickens and wild birds (20, 22). Roy et al. (17) demonstrated that the ITS1 and ITS2 regions are uninformative when focusing at an intraspecific level. For this reason, ITS regions were not sequenced as part of the current study.

Increasing knowledge of genetic diversity and population structure for *D. gallinae* mites from different countries will aid understanding of population structure. These details can support development of alternative strategies for the prevention and treatment of infestations, and support the longevity of new interventions. Previous research based on genetic diversity of the COI gene has focused on *D. gallinae* in parts of Europe, the United States, Brazil, Australia, Japan and South Korea (15, 16, 19, 21). The current study used a combination of phylogeny and network analysis to compare *D. gallinae* COI haplotypes across a broader geographic range in Europe, expanding existing analyses and identifying new COI haplotypes.

## Materials and Methods

### Sample Collection and Distribution

#### United Kingdom

Mites were collected from fifteen farms across the UK from 2017 to 2018, including 11 from England, one from Northern Ireland, one from Wales and two from Scotland. Mites were captured using cardboard traps as previously described (23). Samples were drawn from egg-layer production facilities, with a mixture of free-range (including organic) and enriched cage systems. Mites were either used directly (fresh), dried and frozen at −20°C, or preserved in ethanol (>70% v/v). Up to five individual mites were analyzed from each site to sample mite variants present on each layer farm (Table 1).

**Table 1:** Location of farms sampled in the UK.

**Country**	**County**	**Sample name(s)**	**Number of isolates per farm**	**Production type**
Wales	Cardiganshire	UK 3.1–3.3	3	Free-range
Scotland	Peebleshire	UK 13.1–13.3	3	Intensive
	Highlands	UK 9.0	1	Free-range
Northern Ireland	Tyrone	UK 10.1–10.5	5	Free-range
England	West Sussex	UK 12.1–12.5	5	Free-range
	Kent	UK 5.0	1	Free-range
	Gloucestershire	UK 2.1–2.5	5	Free-range
	Cheshire	UK 4.1–4.4	4	Intensive
	Durham	UK 1.1–1.2	2	Free-range
	Oxfordshire	UK 7.0	1	Free-range
	Shropshire	UK 8.0	1	Intensive
	Suffolk	UK 11.1–11.3	3	Free-range
	Lincolnshire	UK 14.1–14.2	2	Free-range
	Tyne and Wear	UK 15.1–15.2	2	Intensive
	East Sussex	UK 6.0	1	Free-range

#### Mainland Europe

**Figure 1:**
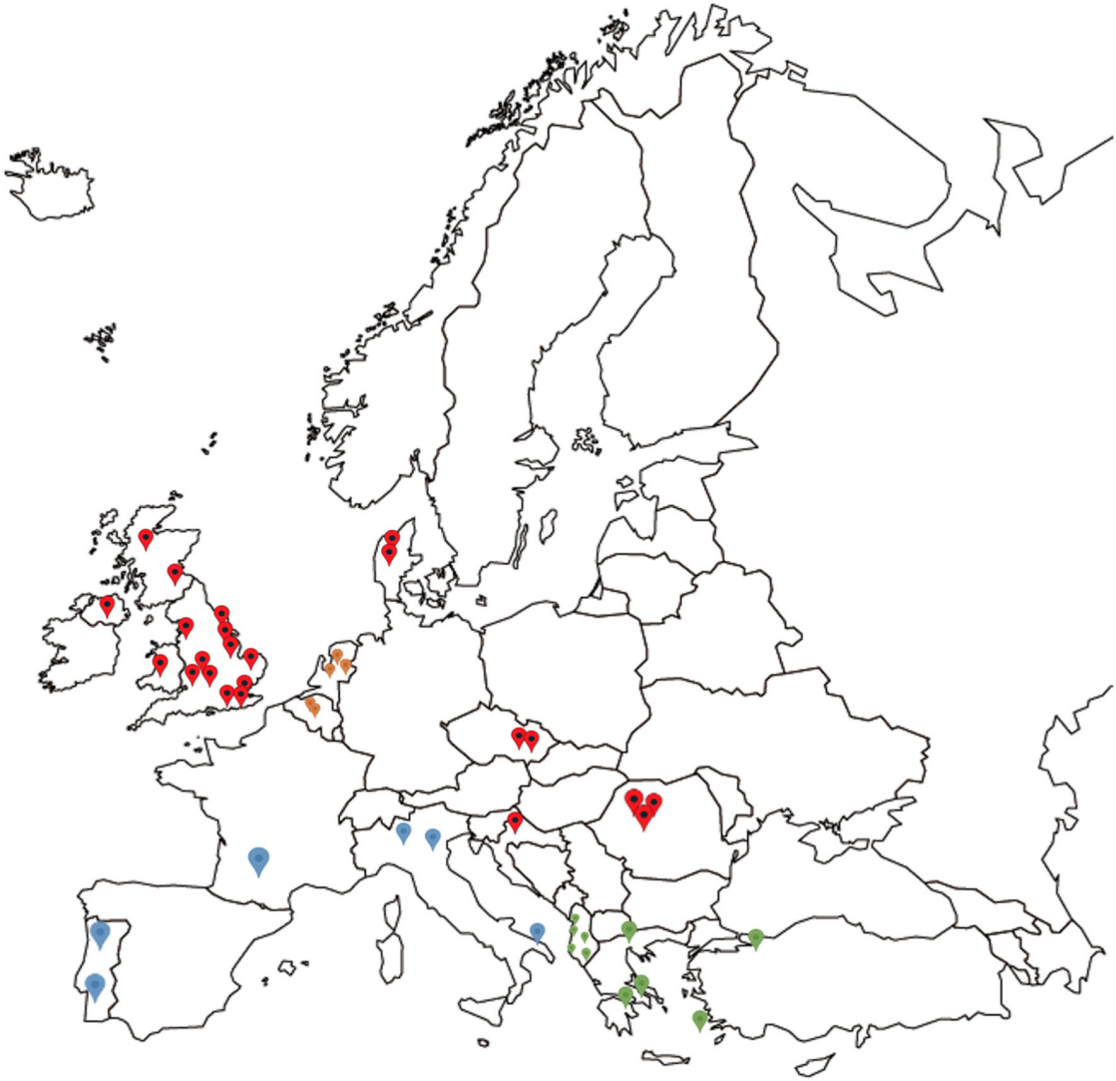
Map showing the origin of all *D. gallinae* populations analyzed in the study, spread across 14 European countries. Red indicating locations for the UK, Czech Republic, Croatia, Denmark, and Romania, blue indicating grouping of France, Portugal, and Italy, green indicating grouping of Greece, Albania, and Turkey, and orange indicating grouping of Belgium and the Netherlands for DNAsp analysis.

Mainland European samples were received preserved in 70–100% (v/v) ethanol or alive in cardboard traps which were either used directly, dried and frozen at −20°C, or preserved in ethanol (>70% v/v). Thirteen mainland European countries were sampled, including Albania (5 farms), Croatia (2), France (2), Portugal (3), Greece (4), Czech Republic (2), Denmark (2), Belgium (2), Romania (5), Turkey (1), Netherlands (5), Italy (5), and Slovenia (3) (Table 2, Figure 1).

**Table 2:** Sample locations from Europe, including the region and number of individual mites sampled.

**Country**	**Number of isolates per country**	**Closest town or region**	**Sample names**	**Number of isolates per region**
Albania	10	Lushnye	ALB1.1, ALB1.2	2
		Berat	ALB2.1, ALB2.2	2
		Korca	ALB3.1, ALB3.2	2
		Peshkopi	ALB4.1, ALB4.2	2
		Durres	ALB5.1, ALB5.3	2
Belgium	8	Destelbergen	BEL1.1–1.6	5
		Evergm	BEL2.1–2.3	3
Croatia	5	Zagreb	CRO1.1–CRO1.5	5
Czech Republic	10	Bohemia	CZH1.1–CZH1.5	5
		South Moravia	CZH2.1–CZH2.5	5
Demark	9	Vejle	DEN1.1–DEN1.5	4
		Jylland	DEN2.1–DEN2.5	5
France	6	Grenade	FRA1.1–1.6	6
Greece	61	Thessaloniki	GRE1.1–GRE1.10	10
		Corinth	GRE2.1–GRE2.13	13
		Leros	GRE3.1–3.25	25
		Attica	GRE4.1–4.13	13
Italy	9	Lecce	ITA1.1–1.3	3
		Varese	ITA2.1–2.2	2
		Verona	ITA3.1–3.4	4
Netherlands	9	Lutten	NET1.1–1.2	2
		Barneveld	NET2.1–2.2	2
		Aalten	NET3.1–3.3	3
		Unknown	NET4.1–4.2	2
Portugal	10	Riveria	POR1.1–POR1.4	4
		Rego	POR2.1–POR2.6	6
Romania	7	Tatarlaua	ROM1.1–1.2	2
		Cuzdrioara	ROM2.2.2–5	4
		Floresti	ROM6	1
Slovenia	7	Tenetiše	SLO1.1–1.3	3
		Škofljica	SLO2.1	1
		Kamnik	SLO3.3–3.5	3
Turkey	6	Karacaali	TUR1.1–TUR1.6	6

### DNA Preparation

Genomic DNA was extracted from 195 individual mites using a Qiagen blood and tissue kit (Qiagen GmbH, Hilden, Germany) according to the manufacturer's protocol, with some modifications. Briefly, mites were homogenized by slicing the whole body with a sterile Agani^TM^ 21G × 1 ¸” (0.8 × 38 mm) needle. The proteinase K digestion step was performed overnight at 56°C and the resulting nucleic acid was eluted in 100 μl. Purified genomic DNA samples were stored at −20°C.

### Polymerase Chain Reaction (PCR)

A 681bp fragment of the *D. gallinae* mitochondrial cytochrome c oxidase subunit I (COI) gene was amplified using the primers COI1Fyuw114 (5′-AGATCTTTAATTGAAGGGGG-3′) and COI1Ryuw114 (5′- AAGATCAAAGAATCGGTGG-3′) corresponding to nucleotide positions 61 to 742 [GenBank accesion number AM921853; (19)].

PCR was perfomed in a volume of 25 μl containing 12.5 μl 2x MyTaq^TM^ (Bioline, London, UK), 400 pM of each primer (Sigma-Aldrich, Darmstadt, Germany) and 2 μl of DNA template. PCR cycling conditions were initial denaturing at 95°C for 5 min, followed by 35 cycles of denaturing at 95°C for 30 s, annealing at 55°C for 30 s, and elongation at 72°C for 30 s. Final elongation was performed at 72°C for 5 min. A T Gradient thermocycler was used (Biometra, Jena, Germany). After amplifcation, PCR products were resolved by electrophoresis in 1.0% (w/v) agarose gels, using 5X DNA loading buffer (Bioline, London, UK), Safeview Nucleic acid stain (NBS Biologicals, Cambridgeshire, UK). The GeneRuler 1 kb ladder (Thermo Fisher Scientific, Waltham, Massachusetts) was used to assess product size. Each PCR amplicon was purified using a Qiagen QIAquick PCR purifiction kit as recommended by the manufacturer (Qiagen GmbH, Hilden, Germany) and eluted in 30 μL dH_2_O.

### Amplicon Sequencing and Analysis

Direct amplicon Sanger sequencing was carried out by Eurofins Genomics employing the same primers as used in the initial reaction. Sequences were assembled and curated using CLC Genomics Workbench 8.1.3 (Qiagen, Aarhus, Denmark). Curated sequences were aligned using CLC workbench 8.1.3 with default parameters and the final alignment was manually curated to detect errors. This resulted in a 565-bp alignment for phylogenetic analysis after low quality sequences were trimmed. Subsequently, model selection for Maximum Likelihood (ML) phylogenetic analysis was determined using MEGA-X (24), identifying the Tamura 3-parameter model. Maximum Likelihood was undertaken with 1,000 bootstrap replicates. Bayesian phylogenetic analysis (MrBayes) was determined using TOPALi v2.5 (25). Model selection identified the Hasegawa-Kishino-Yano (HKY) model with gamma distribution (G) and evolutionary invariable (I). Using the HKY+G+I model, the following parameters were used: 2 runs, 5,000,000 generations and 25% Burnin for construction of a MrBayes tree. Interactive Tree of Life (iTOL) version 4 was used for visualization of MrBayes (26). In parallel, Network 5.0.0.3 (www.fluxus-engineering.com) was used to construct a median-joining (MJ) tree (27). Mites with identical sequences were designated as one haplotype. DNAsp 6.12.03 was used to analyse nucleotide and haplotype diversity (28). All sequences generated here have been submitted to the European Nucleotide Archive under the accession number PRJEB36917.

### Alignment to Genbank Sequences

Nucleotide sequences generated for this study were aligned with published COI amplicon sequences from Japanese *D. gallinae* isolates produced by Chu et al. (19) (Genbank accession numbers: LC029457-LC029557), creating an alignment of 554-bp. These sequences were used due to utilization of the same forward and reverse primers.

## Results

### Nucleotide Sequence Analysis

In total 195 COI sequences were obtained from mites collected from 14 European countries representing 82 farms (European Nucleotide Archive accession no.s PRJEB36917). A 565bp alignment representing a fragment of the *D. gallinae* mitochondrial COI gene was analyzed. The nucleotide frequences were 29.05% (A), 40.62% (T), 14.65% (C), and 15.67% (G). Two countries were represented by more than 30 sequences; Greece and the UK. Comparison of nucleotide alignments for each of these countries and the full dataset revealed higher nucleotide and haplotype diversity in the UK comparatively to Greece. Haplotype diversity was similar for the full dataset and the UK (0.917 and 0.901, respectively) whilst observably lower for Greece (0.521) (Table 3).

**Table 3:** Nucleotide diversity, average number of nucleotide differences and haplotype diversity for the full dataset and individual countries.

**Samples**	**Nucleotide diversity (per site), Pi**	**Average number of nucleotide differences, k**	**Haplotype (gene) diversity**
All samples	0.02560	14.38598	0.917
UK	0.01403	7.84480	0.901
Greece	0.00419	2.36831	0.521
Albania	0.02124	11.97778	0.889
Belgium	0.01991	11.25000	0.964
Denmark	0.01517	8.556	0.861
Croatia	0.00319	1.8000	0.900
Czech Republic	0.01529	8.62222	0.933
Denmark	0.01517	8.55556	0.861
France	0.00153	0.86667	0.733
Italy	0.00345	1.94444	0.722
Portugal	0.02191	12.37778	0.889
Romania	0.00405	2.28571	0.286
Slovenia	0.02630	14.85714	0.857
Turkey	0.00059	0.33333	0.333
Netherlands	0.02557	14.4444	0.944
Greece, Albania, and Turkey	0.01182	6.66439	0.695
Portugal, France, and Italy	0.01297	7.31333	0.877
Belgium and the Netherlands	0.02439	13.77941	0.963

Overall, for nucleotide diversity and the average number of nucleotide differences, the lowest scores were observed in Turkey and the highest in Slovenia, whilst the lowest haplotype diversity was seen in Romania and the highest in Belgium.

### Variation in the United Kingdom

A total of 39 COI sequences were obtained from the UK, one of the most intensively sampled countries with the largest number of independent 15 farms. Alignment revealed 27 mutations between samples when compared to the consensus (Table 4). Out of these 27, eight were found to represent a single farm, seven an individual country within the UK, and six were detected in a single isolate. No insertions or deletions were seen. Out of the 15 farms sampled, five were represented by a single sequenced isolate and as such were not included in the intra-farm analysis. There was an even split amongst the remaining ten farms, with five demonstrating intra-farm sequence variation and five showing no intra-farm variation (including Northern Ireland). Twenty six of the 27 mutations found in the UK had at least one farm with intra-farm variation, with the exception of one mutation found only in all Irish isolates (Tables 4, 5). At seven nucleotide sites, only one of the five farms showed variation, with three of these from a single farm (UK15).

**Table 4:** Variable positions for UK isolates in comparison to the consensus.

**Base pair position relative to alignment**	**Consensus**	**Mutation**	**No. of individuals consensus**	**No. of individuals with mutation**	**Mutation found from a single country**
9	A	G	34	5^*^	Northern Ireland
33	T	C	38	1^*^	Scotland
36	C	T	24	15	–
37	T	C	25	14	–
60	T	C/A	37	1/1^*^	Wales/England
69	A	G	24	15	–
123	A	G	36	3	–
126	A	G	24	15	–
154	T	C	37	2	–
162	T	A	24	15	–
167	C	T	38	1^*^	England
174	A	G	36	3	–
189	C	T	21	18	–
300	T	C	34	5	–
336	T	C	24	15	–
360	A	G	28	11	–
396	T	C	24	15	–
411	C	T	24	15	–
450	G	A	26	13	–
456	T	C	34	5	–
465	C	T	38	1^*^	England
480	A	T	38	1^*^	England
498	T	C	37	2	–
528	T	C	21	18	–
534	A	G	27	12	–
546	T	C	38	1^*^	England
549	G	A	38	1^*^	England

* *All isolates belonging to a single farm*.

**Table 5:** Intra-farm variation observed in farms from the UK.

**Base pair position**	**Farm**	**Country**	**Consensus**	**Mutation**	**No. of individuals consensus**	**No. of individuals with mutation**	**Total no. of individuals**
33^*^	UK13	Scotland	T	C	1	2	3
36	UK14	England	C	T	1	1	2
	UK3	Wales			1	2	3
37	UK2	England	T	C	2	3	5
	UK3	Wales			1	2	3
60	UK3	Wales	T	C	1	2	3
	UK15	England	T	A	1	1	2
69	UK3	Wales	G	A	1	2	3
	UK14	England			1	1	2
123	UK13	Scotland	A	G	1	2	3
	UK14	England			1	1	2
	UK15	England			1	1	2
126	UK3	Wales	A	G	1	2	3
	UK14	England			1	1	2
153	UK13	Scotland	T	C	1	2	3
	UK14	England			1	1	2
162	UK3	Wales	T	A	1	2	3
	UK14	England			1	1	2
167^*^	UK11	England	C	T	1	2	3
174	UK11	England	A	G	1	2	3
	UK13	Scotland			2	1	3
189	UK3	Wales	T	C	2	1	3
	UK14	England			1	1	2
300	UK3	Wales	T	C	1	2	3
	UK14	England			1	1	2
336	UK3	Wales	T	C	1	2	3
	UK14	England			1	1	2
360	UK3	Wales	A	G	1	2	3
	UK11	England			2	1	3
	UK15	England			1	1	2
396	UK3	Wales	T	C	2	1	3
	UK14	England			1	1	2
411	UK3	Wales	C	T	2	1	3
	UK14	England			1	1	2
450^*^	UK2	England	G	A	3	2	5
456	UK3	Wales	T	C	2	1	3
	UK14	England			1	1	2
465^*^	UK15	England	C	T	1	1	2
480^*^	UK15	England	A	T	1	1	2
498	UK13	Scotland	T	C	2	1	3
	UK14	England			1	1	2
528	UK3	Wales	T	C	2	1	3
	UK14	England			1	1	2
546^*^	UK11	England	T	C	2	1	3
549^*^	UK15	England	G	A	1	1	2

* *All variants collected from a single farm*.

### Intra-farm Variation: Greece

A total of 51 COI sequences were obtained from Greece, the most intensively sampled country, providing a second opportunity, alongside the UK, to look at intra-farm variation. Intra-farm variation was detected in mites from all four Greek farms at five nucleotide positions at variable rates, with between 20 and 68% of samples from a single farm presenting the mutation in comparison to the consensus.

### Phylogenetic Analysis of Sequences Generated in This Study

**Figure 2:**
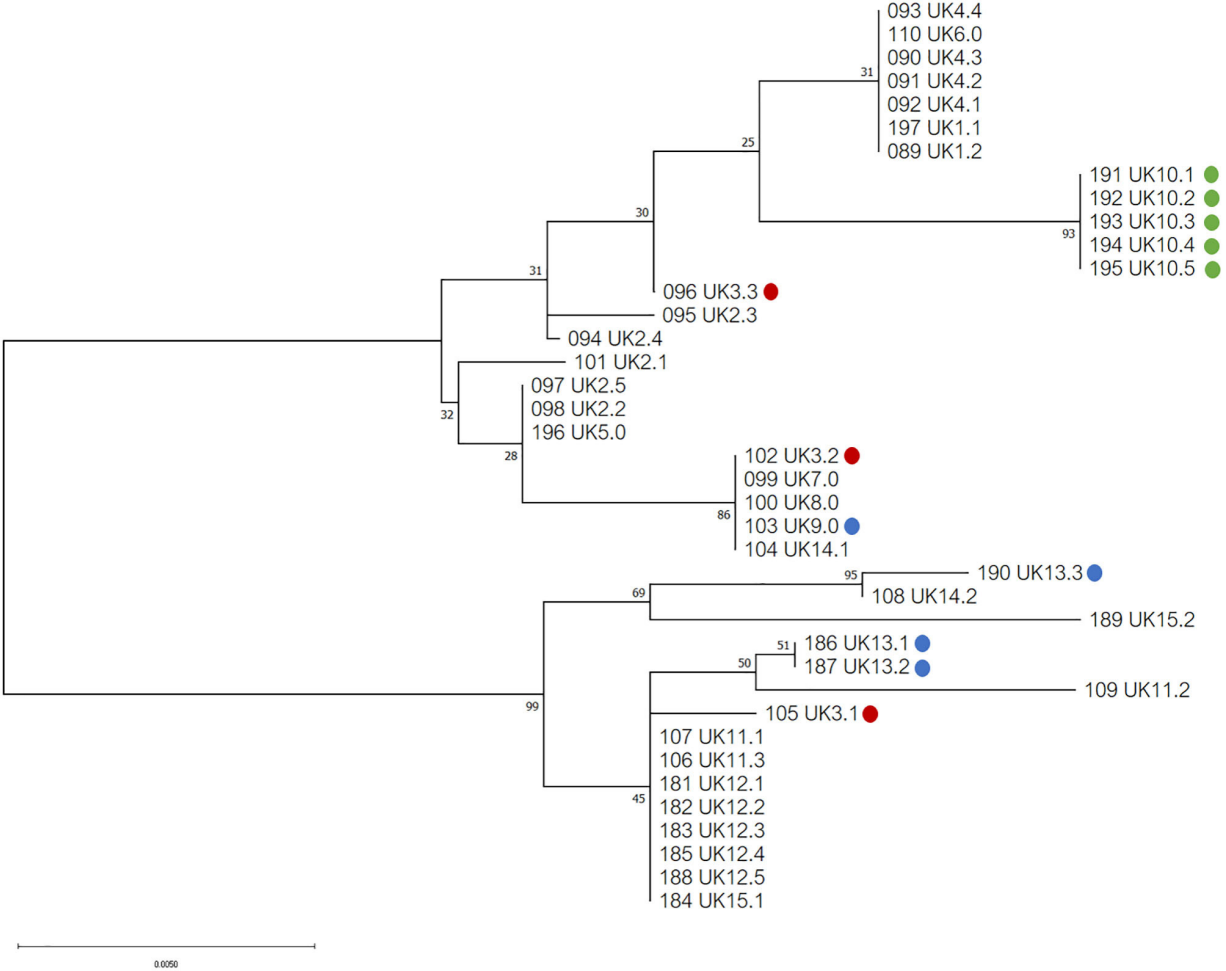
Phylogenetic tree of UK isolates inferred using the Tamura 3-parameter and maximum-likelihood (29). A discrete Gamma distribution was utilized to model evolutionary differences among sites [5 Categories (+G, parameter = 0.0500)]. A total of 565 positions were used in the analysis, encoding 39 nucleotide sequences. All evolutionary analysis was completed with MEGA X (24). Countries from the UK are indicated as follows: England = no color, red = Wales, blue = Scotland, green = Northern Ireland.

Phylogenetic analysis of the 39 COI sequences from the UK revealed two major haplogroups, with a total of seventeen haplotypes (Figure 2). Ten haplotypes were located in haplogroup one, with seven haplotypes in haplogroup two. At a country level, Northen Ireland grouped into one haplotype (haplogroup one), which was not shared with England, Scotland or Wales, although all isolates came from a single Northern Irish farm. Isolates from Scotland, England and Wales were found distributed in both haplogroups but only one haplotype shared isolates from all three countries.

**Figure 3:**
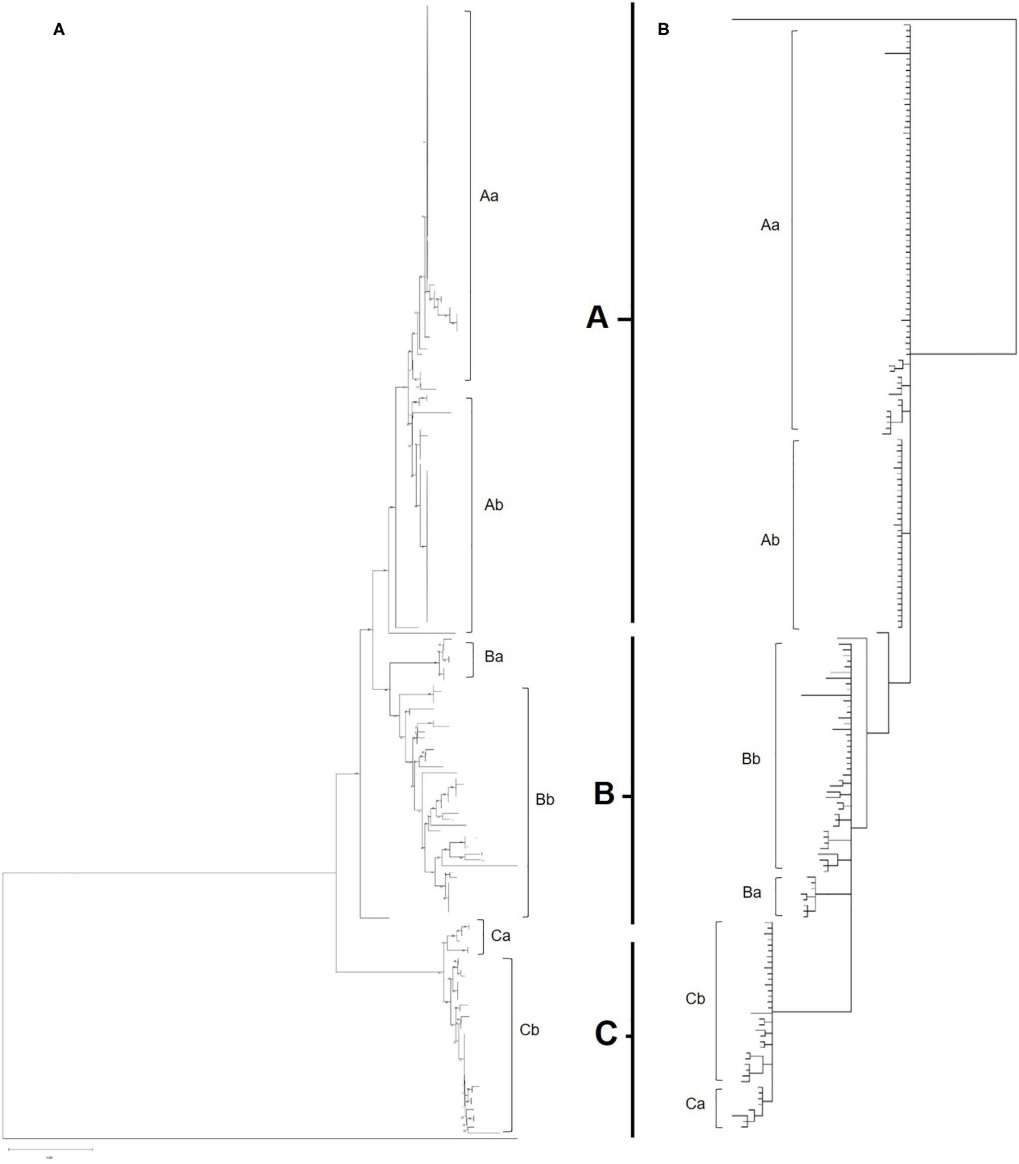
**(A)** Phylogenetic tree of all European and UK isolates sequenced as part of this study. Inferred using the Tamura 3-parameter and maximum-likelihood with 1,000 replicates (29). A gamma distribution was utilized to model evolutionary differences (shape parameter = 0.5). A total of 565 positions were used in the analysis, encoding 196 nucleotide sequences. All evolutionary analysis was completed with MEGA X (24). **(B)** Bayesian phylogenetic tree of all European and UK isolates sequenced as part of this study. Inferred using the HKY+G+I model with 2 runs, 5,000,000 generations and 25% Burnin. A total of 565 positions were used in the analysis, encoding 196 nucleotide sequences. Evolutionary analysis completed on TOPALi (25) and edited on iTOL (26).

Maximum likelihood phylogenetic analysis of the complete set of 195 COI sequences revealed 76 distinct haplotypes that clustered into three main haplogroups: A, B, and C (Figure 3). Group A consisted of 22 haplotypes from 10 countries, group B 34 haplotypes from seven countries and group C 20 haplotypes from seven countries. The three major haplogroups diverged into a further six sub-lineages designated as Aa, Ab, Ba, Bb, Ca, Cb (Figure 3). Group Aa included 14 haplotypes, group Ab nine haplotypes, group Ba four haplotypes, group Bb consisted of 30 haplotypes, group Ca four haplotypes and Cb consisted of 16 haplotypes. In total, sequences from 8 out of 14 countries clustered into a single haplogroup, 4 out of 14 countries into two haplogroups and 2 out of 14 countries into three haplogroups.

**Figure 4:**
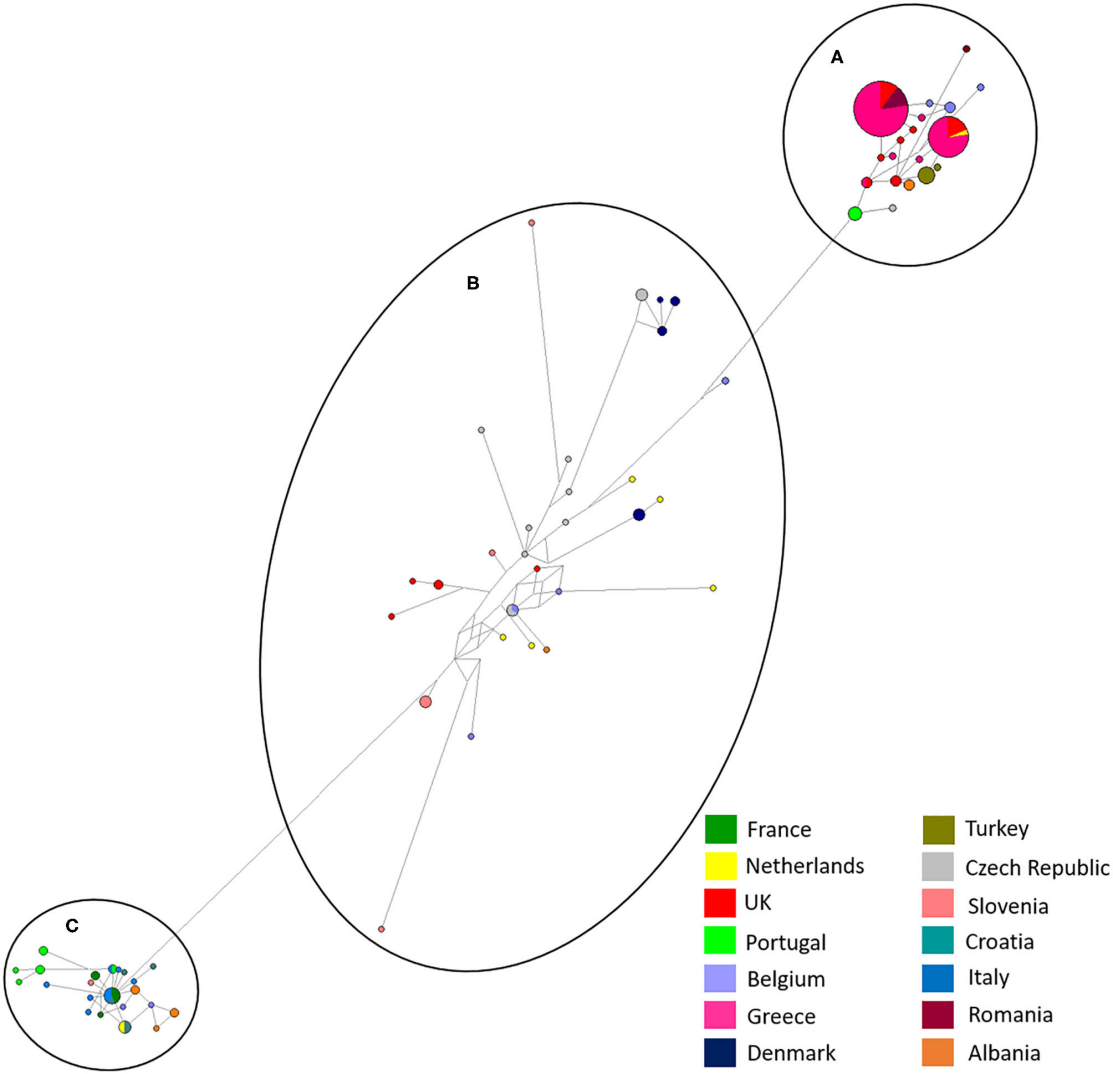
Network analysis of all European isolates sequenced in the study with the three main haplogroups labeled, **(A–C)**. A total of 565 positions were used in this analysis, encoding 195 nucleotide sequences. Color coded key provided for country identification.

Albania and the Netherlands were the only two countries where *D. gallinae* isolates were represented by sequences from all three haplogroups. Isolates from Greece and Romania were only found in sub-groups Aa and Ab and Turkish isolates were only found in sub-group Ab (two haplotypes), but it should be noted that only one farm from Turkey was sampled. Denmark was the only country to be found solely in sub-groups Ba and Bb, representing three out of the four haplotypes found in sub-group Ba. Sub-haplogroup Ca was the only subgroup to represent a single country, entirely consisting of six *D. gallinae* isolates collected across three farms from Portugal. The remaining four Portugese isolates were clustered into subgroup Aa (three) and Cb (one). The main haplogroups identified in the phylogenetic tree can be observed in Figure 4. Turkey and Romania were the only countries to have just two haplotypes. Five farms from Romania were sampled, all located in haplogroup A. Four farms clustered in one haplotype and the remaining farm in a single haplotype.

Bayesian phylogenetic analysis supported topology from ML with three main haplogroups: A, B, and C that diverge into six subgroups: Aa, Ab, Ba, Bb, Ca, Cb (Figure 3). Variation in the order of individual haplotypes within subgroups was observed when comparing ML and MrBayes trees (Figure 3), but overall tree topology remained consistent. Identical clustering of countries in haplogroups was observed; eight clustering in a single haplogroup, four in two haplogroups and two in three haplogroups (Albania and the Netherlands).

### Comparative Analysis With GenBank Sequences

**Figure 5:**
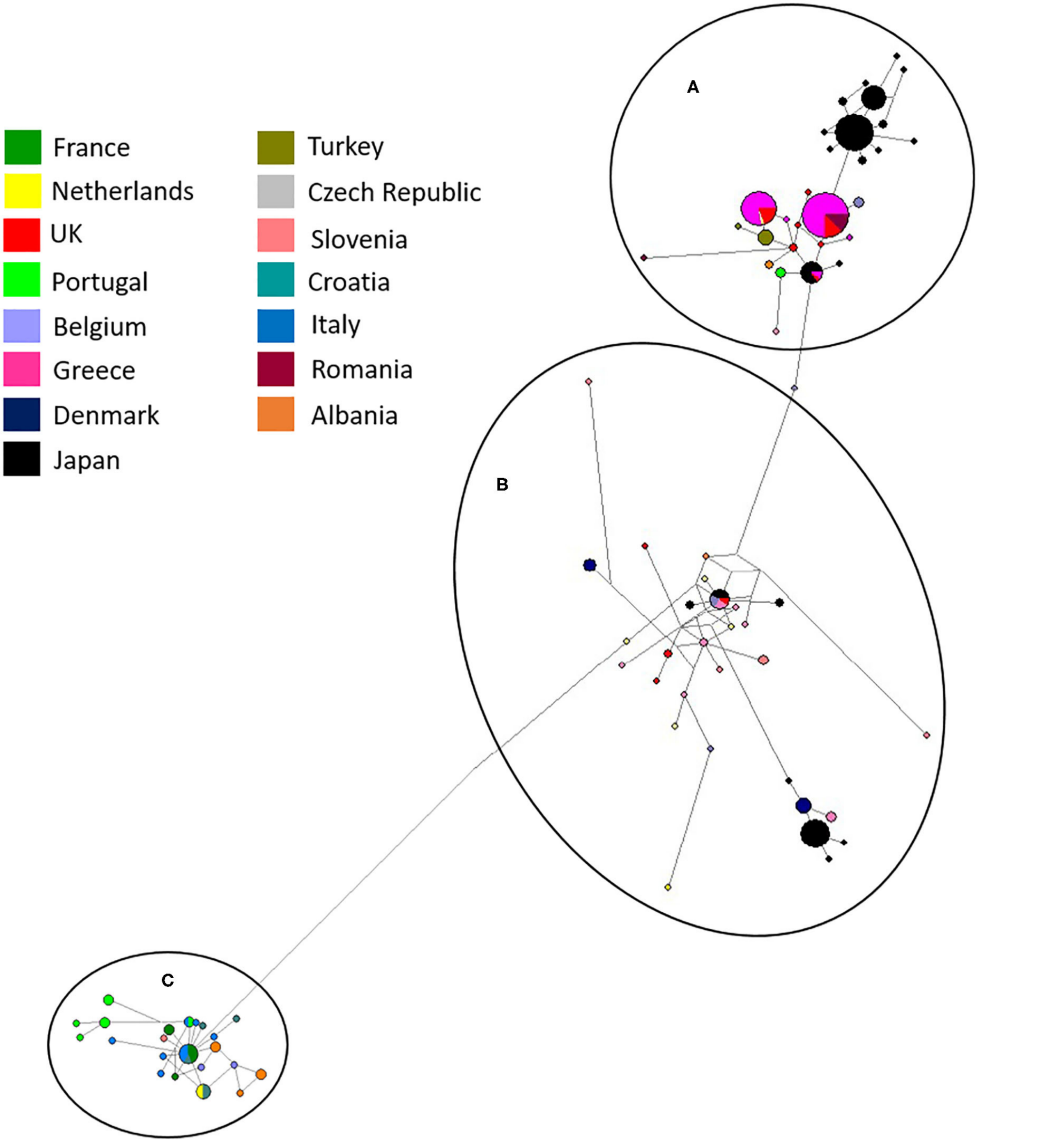
Network analysis of all European and UK isolates sequenced in the study and Japanese sequences available from Genbank (19). The three main haplogroups are labeled **(A–C)**. A total of 554 positions were used in this analysis, encoding 270 nucleotide sequences. Color coded key provided for country identification.

Network analysis confirmed that European and Japanese samples were genetically related, as previously demonstrated (19). One haplotype was common to Japan, UK (England) and Greece in haplogroup A (Figure 5). In haplogroup B, another haplotype was common to Japan, Belgium, the Czech Republic and the UK (Figure 5). Network analysis comparing Japanese and UK isolates showed three main haplogroupsOne consisted purely of Japanese samples, including one dominant haplotype, and two further haplogroups contained a mixture of Japanese and UK haplotypes. England was the only country found to directly share haplotypes with Japan. A total of three shared haplotypes are seen, two made up mostly by Japanese isolates and one more common to English isolates. No shared haplotypes were observed between Japan, Wales, Northern Ireland or Scotland, although all five countries were found clustered in haplogroup two.

## Discussion

In this study, the phylogeny of *D. gallinae* populations was assessed through sequencing of mitochondrial COI gene amplicons from 82 farms spread over 13 mainland European countries and the United Kingdom, including seven countries not previously studied. Previous research focusing on COI diversity in *D. gallinae* has demonstrated multiple lineages with comparative analysis concluding that cryptic species must be present (15, 28, 30). In the present study, multiple lineages were found during phylogenetic analysis with three main haplogroups (A, B, C), supported by both ML and Bayesian phylogentic analyses (Figure 3) The C group haplotypes branched earlier in the phylogenetic tree when compared to groups A and B. in some cases, as one might expect, clustering between countries sharing a border or located closely geographically can be seen This is demonstrated in haplogroup C where sequences from Italy, Croatia, Albania, France, Slovenia, the Netherlands, and Portugal have clustered. DNAsp analysis showed variation in nucleotide and haplotype diversity when looking at countries grouped by geographic distance (Figure 1). Analysis focusing on the Netherlands and Belgium demonstrated high nucleotide diversity, close to that observed for the full dataset, as well as greater haplotype diversity. Conversely, groupings of Albania, Greece and Turkey, and Portugal, France, and Italy both showed a lower nucleotide and haplotype diversity (Table 3). Samples spread across a greater geographical distance (e.g., Denmark to Slovenia) are seen clustered in haplogroup B. Network analysis illustrated the occurrence of shared haplotypes between multiple European countries (e.g., Belgium and the Czech Republic in Figure 4) and in conjunction with comparative analysis between UK, mainland European, and Japanese samples (Figure 5) supports previous evidence of international and intra-national movement of mites (19). For future investigation, data obtained from connecting countries (i.e., Spain, Bosnia and Herzegovina and Montenegro) would aid in developing a clearer picture. At present, it does not seem feasible to predict *D. gallinae* diversity based on gegraphical location with phylogenetic analysis in this study demonstrating instances of geographical clustering, geographic diversity but also non-geographical clustering.

Establishment of *D. gallinae* populations from limited numbers of individuals is anticipated to have consequences on the level of genetic diversity. Expansion from a limited number of mites is likely to result in a relatively smaller number of haplotypes than expansion from a larger number of mites (16). Focusing on the UK, it was clear that despite being a group of islands the mite populations sampled were genetically related to those found in mainland Europe and Japan with nine haplotypes spread through haplogroups A and B (Figure 3) and three shared haplotypes between England and Japan (Figure 5). Identical sequences were found in one haplotype originating from the UK, Japan, Belgium and Czech Republic, and in another haplotype from the UK, Japan and Greece. It seems most likely that trade between countries, either historical or on-going, provides an opportunity for admixture between countries allowing for shared haplotypes to be seen. Comparing UK farms, it is interesting to note that only 50% of farms showed intra-farm variation, suggesting that some farms host limited population diversity. However, it is worth noting that this could have been related to low numbers of mites sampled per farm. Similarly, expanding the analysis to additonal loci might have identified further genetic diversity. At this point, there appears to be no link between production system and intra-farm variation, with both free-range and caged systems found in both categories.

Intra-farm variation was observed in all four of the Greek farms sampled (Table 6), where three farms (Thessaloniki, Leros and Attica) had three haplotypes and one farm (Corinth) had two haplotypes. All of the haplotypes were assigned to haplogroups Aa and Ab and the two haplotypes from Corinth were shared by all three other farms (Figure 3). These two haplotypes represent the majority of isolates sampled from Greece, totalling 58 of 61. However, the third haplotype for Leros, Thessoliniki and Attica was individual to each farm, and, interestingly, shared an identical sequence with an isolate originating in the UK (Figure 3). Similar results were demonstrated for two farms investigated by others in Norway, where two and three different haplotypes were discovered from 17 to 19 individual *D. gallinae*, respectively (16). These authors reasoned that multiple haplotypes in a single farm is indicative of the farms either being infected by multiple haplotypes or experiencing multiple infections, stating that mite populations with contact have an increased chance of shared haplotypes than those with barriers separating them. In cases where haplotype occurrence cannot be explained by geographical location they likely result from contaminated equipment, infected chickens or other materials being moved between farms. The scattering of haplotypes found in the present study are suggestive of the latter being true, that shared haplotypes could result from infected chickens or materials. Three of the farms sampled were located on the Greek mainland and the final farm was located on Leros, one of the islands in the Aegean sea. Despite being separated by the Aegean sea, all four farms shared two haplotypes, suggesting a common original source for all farms or continuous admixture between them. That would be possible by transport or trade routes or sharing of contaminated equipment. This is also exemplified when considering that the common haplotype for all Greek farms found in haplogroup Aa also contained isolates from the UK.

**Table 6:** Intra-farm variation from Greek farms for five nucleotide positions.

**Base pair position**	**Farm**	**Consensus**	**Mutation**	**No. of individuals consensus**	**No. of individuals with mutation**	**% of individuals with mutation**	**Total**
42	THE	T	A	3	7	70%	10
	LER			8	17	68%	25
	ATT			6	7	54%	13
	COR			7	6	46%	13
305	THE	T	C	8	2	20%	10
	LER			18	7	28%	25
	ATT			9	4	31%	13
	COR			7	6	46%	13
455	THE	A	G	8	2	20%	10
	LER			17	8	32%	25
	ATT			8	5	38%	13
	COR			6	7	54%	13
461	THE	T	C	10	2	20%	10
	LER			17	8	32%	25
	ATT			7	6	46%	13
	COR			6	7	54%	13
539	THE	A	G	3	7	70%	10
	LER			17	8	32%	25
	ATT			6	7	54%	13
	COR			7	6	46%	13

## Conclusions

This study provides evidence for genetic diversities in *D. gallinae* distributed across Europe. Where sufficient sequence depth was generated intra-farm variation was detected in the United Kingdom and Greece. In addition, phylogenetic analysis provided further support for international and intranational movement of *D. gallinae*. Mapping additional COI diversity in countries not yet researched would help to build a more comprehensive understanding.

## Data Availability Statement

All sequences generated here have been submitted to the European Nucleotide Archive under the accession number PRJEB36917.

## Author Contributions

DB, FT, and AN designed the project and obtained funding. EK-T led the experimental work, with support from DB, TK, EP, and AA. EK-T led analysis, with support from DX and DB. EK-T drafted the manuscript, with contributions from TK, AA, EP, AN, DX, FT, and DB. All authors contributed to the article and approved the submitted version.

## Conflict of Interest

The authors declare that the research was conducted in the absence of any commercial or financial relationships that could be construed as a potential conflict of interest.
